# “Doing extra work and not getting extra help”: the burden of work generated to manage the “no visitors” policy during the COVID-19 pandemic in Ontario, Canada

**DOI:** 10.3389/fpubh.2025.1632828

**Published:** 2025-09-16

**Authors:** Danielle Jacobson, Arija Birze, Elizabeth Mansfield, Walter P. Wodchis, Terence Tang, Mehdi Ammi, Sara J. T. Guilcher, Kerry Kuluski

**Affiliations:** ^1^Institute for Better Health, Trillium Health Partners, Mississauga, ON, Canada; ^2^Institute of Health Policy, Management, and Evaluation, University of Toronto, Toronto, ON, Canada; ^3^Department of Occupational Science and Occupational Therapy, University of Toronto, Toronto, ON, Canada; ^4^Department of Medicine, University of Toronto, 6 Queen’s Park Crescent West, Toronto, ON, Canada; ^5^School of Public Policy and Administration, Carleton University, Ottawa, ON, Canada; ^6^Department of Physical Therapy, Temerty Faculty of Medicine, University of Toronto, Toronto, ON, Canada; ^7^Leslie Dan Faculty of Pharmacy, University of Toronto, Toronto, ON, Canada

**Keywords:** patient- and family-centred care, caregivers, cancer-care, healthcare team, delayeddischarge, alternative level of care, COVID-19, pandemic

## Abstract

**Background:**

During the pandemic, a “no visitors” policy was implemented across hospitals in Ontario, Canada. Without caregivers present in-hospital to support patient care (e.g., treatment decision-making, advocacy, treatment compliance, social support), there was a perceived decline in care quality. Despite existing research on the extra work required to navigate the loss of caregiver support in-hospital, there is a paucity of understanding about the work required to manage the “no visitors” policy itself—including creative ways to work around it. This qualitative research study draws attention to the “no visitors” policy and the work to manage and work around these limitations across healthcare system silos (drawing on cancer care and alternate level of care as case examples) in Ontario, Canada.

**Methods:**

In total, 5 focus groups and 53 interviews were conducted with 68 participants (10 patients, 7 caregivers, 40 healthcare providers, and 11 healthcare decision-makers). The authors engaged in codebook thematic analysis.

**Findings:**

Managing the “no visitors” policy and pushback against it generated a significant burden of work for patients, caregivers, healthcare providers, and healthcare decision-makers at a time when difficult emotions were high and resources and capacity were low. Five themes are discussed that depict the burden of work: (1) work of making individual exceptions to the “no visitors” policy, (2) work of standardizing exceptions, (3) work to remedy and navigate inconsistencies across hospital units or partner organizations, (4) workarounds to gain in-hospital entry via “hot words” and sneaking in, and (5) workarounds when in-hospital entry was not possible via technology and visiting through windows.

**Conclusion:**

The denial of caregivers’ entry into hospitals during the COVID-19 pandemic undermined their value as essential care partners, despite their contributions to patient care. Unintended consequences of such public health policy, including the generation of burdensome work to manage and work around it for all involved, must therefore be more carefully considered for future pandemic preparedness.

## Introduction

Prior to the COVID-19 pandemic, many hospitals in Canada had implemented open visitation policies (1), indicating that support from and integration of caregivers was central to person- and family-centered care (2, 3). With cancer care in particular, caregivers ensured “treatment compliance, continuity of care, and social support” (4 p. 145, 5). More specifically, caregivers played a pivotal role in treatment decision-making and advocacy, with patients often refraining from making care-related decisions without a caregiver’s input (6, 7). Caregivers also played a crucial role in care transitions. For example, when patients were stuck in transition from hospital to their next point of care [e.g., referred to as delayed discharge or ALC (8, p. 1)], caregivers often contributed support with bathing, meals, and ambulation (9), in addition to providing valuable understanding for healthcare teams on individual patient needs (10).

The authors define “caregivers” as any family or chosen family (e.g., those with a familial-like or strong bond who may not bear biological or legal relation) and friends who provide formal or informal, paid or unpaid support to patients navigating healthcare. The COVID-19 pandemic period is defined as beginning in early 2020 with no official end date given the lasting and continuing impact within the healthcare context. In this paper, the authors particularly discuss the time point during the pandemic when “no visitors” restrictions were applied.

Despite the demonstrated importance of caregivers to healthcare prior to the COVID-19 pandemic, shortly after its onset, “no visitors” restrictions were implemented across Ontario to address the state of pandemic-related emergency in March 2020 (11). The term ‘“no visitors” policy’ is mobilized in this work given that it was the term used by participants in this study. The “no visitors” policy was originally mandated by the Ontario provincial government when there was a lack of knowledge about the infection rate, transmissibility, and health-related consequences of COVID-19. Understandably, the intention was to protect those not already infected and preserve limited resources such as personal protective equipment (12). However, these measures were not necessarily evidence-based nor collaborated on with patient and caregiver partners and organizations (12, 13). Tam et al. (14) particularly point to directions from the Chief Medical Officer of Health to limit “visitors” in hospitals followed by the generation of a “standardized approach” to implementation by the Toronto Region COVID-19 Hospital Operations Table (p. 30).

The “initial visitor access policy,” as described by the table, allowed entrance only to those who were “deemed essential in specific circumstances” [e.g., for youth, for someone who is dying (15, p. 1)]. The resultant standard guidance (and what is referred to by the term ‘“no visitors” policy’) across a plethora of sources was therefore to limit individuals gaining entry into hospital sites [e.g., Chief Medical Officer of Health of Ontario (16), Ontario Health Toronto (15), Ontario Ministry of Health (17, 18)]. For example, the Ontario Ministry of Health (17) detailed guidance for navigating “no visitors” restrictions, which have since been incorporated into broader guidance for response to infectious diseases (18). This guidance includes avoidance of “non-essential visits to individuals who are immunocompromised or at higher risk of illness … [or] to high risk settings such as hospitals [for those who] have COVID-19 symptoms” or are close contacts of those with symptoms (18, p. 11). Nonetheless, the document was for recommendation purposes and was not “intended to take the place of medical advice … legal advice” nor “directives issued by the Minister of Health or the Chief Medical Officer of Health” (17, p. 1). Ultimately, while mandated by the government, implementation was left to organizations themselves, particularly managers and front-line teams, to interpret and implement the policy.

While the “no visitors” policy was meant to conserve resources and protect those in-hospital from the spread of COVID-19 (13, 19), its instatement implied that caregivers, despite being previously labeled as “essential partners in care” (20 p. 2., 21), were not really considered a vital component of the healthcare team in many contexts (22). With limited caregivers present in-hospital to support patient care, patients often missed care-related information, lacked portering and translation support, and had insufficient assistance with basic necessities [e.g., going to the bathroom, eating post-surgery (22)]. Patients also suffered from a paucity of mental health support [a core element of recovery (23)] in addition to loneliness, anxiety, and isolation (24). These challenges were present especially during in-hospital appointments (25), with a noted intensified effect on older adults (26). Some healthcare providers understood the protective intentions behind the restrictions but ultimately felt that restricting caregivers from supporting patients in person was “antithetical” to person- and family-centered care and their professional “ethic of family togetherness” (27, p. 5).

Without caregivers in person, there was a lack of opportunity to build trust between patients, caregivers, and healthcare providers, and a diminished ability to gain knowledge through observation, whether it be for caregivers regarding their loved one’s condition or for providers regarding caregivers’ concerns (28). It is now generally understood that strict visitor restrictions during the pandemic negatively impacted the physical and psychosocial health of all stakeholders, creating additional demands for care and ethical tensions for healthcare providers (29–31). Therefore, there may be an opportunity to grapple with traditional models for thinking about the ethical justification for visitor restrictions, incorporating lessons learned from the COVID-19 pandemic, taking into account patient and caregiver perspectives, and considering proportionality, impact for stakeholders, cultural considerations, and harm mitigation (32).

Despite the existing research that depicts the extra work that became foisted onto all involved in order to navigate the loss of caregiver support as a result of the “no visitors” policy during the COVID-19 pandemic, there is a paucity of understanding about the work required to manage the policy itself. Notably, there is scant reflection in the literature from previous pandemics on the impact of “no visitors” restrictions on patients, caregivers, families, and healthcare providers. Reflection from the 2003 SARS and 2009 swine flu (H1N1) pandemics instead focuses on, for example, insufficient hospital preparedness (33), mitigation of communicable disease, healthcare worker safety (34), and screening prior to hospital entry (35). The current research thus aims to garner a better understanding of the added burden of work to implement, enforce, maintain, modify, and work around the “no visitors” policy. This evidence will be useful to inform future public health recommendations for similar policies during times of infection prevention and control crises.

## Methods

### Aims and scope

The authors carried out two independent studies, each with its own ethical provisions, exploring the impacts of the COVID-19 pandemic on the experiences of patients, families, caregivers, and healthcare professionals engaged in different types of care in Ontario hospital systems. Over the course of conducting these pandemic-specific impact studies, it became clear through analytic discussions that there were several points of convergence in the work with respect to the broadly applied “no visitors” restrictions *across* healthcare system operations. This prompted the authors to combine analyses from the two studies in order to provide a more nuanced, wider breadth of understanding. At first, findings were explored separately and then converged when stark similarities were observed. Therefore, a secondary analysis was not carried out; rather, the primary analysis done by the same research team from the two studies was combined due to the similarities. Each study received REB approval, and all measures were in place and followed to maintain confidentiality. One study centered on the impacts of the COVID-19 pandemic on patients designated with a delay in hospital discharge, known in Canada as alternate level of care (ALC), and the other investigated cancer care experiences. The two studies were conducted in parallel under the same research portfolio.

The cancer care project was guided by the research question: What were cancer patient, caregiver, healthcare provider, and healthcare decision-maker experiences during the COVID-19 pandemic? The aim was to garner knowledge surrounding the wider impact of the COVID-19 pandemic on cancer care in Ontario, Canada. What began as questions surrounding delays in care and access to care evolved into discussions about the “no visitors” policy, which participants indicated was important to them and had a significant impact on their experiences.

The ALC transitions in care project was guided by the research question: What are the experiences of ALC patients, caregivers, healthcare providers, and decision-makers during the COVID-19 pandemic? What are the mitigation strategies to address the impacts of transitions in care? The aim was to better comprehend the experiences of ALC patients who were transitioned to other hospital units, hospitals, or out-of-hospital facilities (e.g., home and long-term care) during the COVID-19 pandemic in Ontario, Canada. Glaringly clear was participants’ inclination to discuss the “no visitors” policy and the ways in which it impacted their experiences and ability to carry out their work. Therefore, the research question guiding the analysis in this manuscript was: How was the “no visitors” policy managed during the COVID-19 pandemic?

### Conceptual framework and reflexivity

A critical-social, social-justice-oriented approach guided the research, given the goal to better understand current strengths and growth opportunities in cancer care and ALC transitions in care for health system improvement (36). The epistemology underlying the inquiries, which influenced the authors’ approach to participant interactions, data collection, analysis, and knowledge dissemination, surrounded their view of knowledge as value-mediated and experiences as shaped by institutional structures [e.g., policies, social discourse, training (37, 38)]. Also underlying the way in which the research was carried out was the varying positionality of each research team member (39, 40). Research expertise spanned across multiple disciplines, including qualitative health research, applied health services research, cancer research, aging and long-term care research, ALC research, patient and caregiver experience research, and women’s health research. Researcher experience also ranged from early to mid- and senior-career stages. Team members had a range of racial and ethnic intersections, and some had direct experience as a caregiver to a patient with cancer and to a patient designated with ALC status receiving care in Ontario, Canada.

### Recruitment and consent

Purposive recruitment for the cancer care study took place from February 2023 to May 2024 at two hospital sites in Ontario, Canada, to capture the perspectives of the diverse and often marginalized communities served by each location. Both inpatient and outpatient care were included. Comprehensive cancer care was available at each hospital site (e.g., inpatient and outpatient treatment, diagnostic and surgical services, cancer treatment, mental health, and other supportive services). Recruitment for the ALC study took place from January to August 2023 across four hospital sites in Ontario, Canada (two of which overlapped with hospital sites involved in the cancer care study). Inpatient acute and post-acute sites were included, incorporating the experiences of patients and caregivers with diverse care pathways and destinations.

For both studies, potential patients and caregivers received recruitment flyers via mail or email, facilitated by staff who did not provide direct care to the recipients. Flyers were also posted in hospital waiting rooms. Healthcare providers and decision-makers were recruited with the help of collaborators across the hospitals who dispersed recruitment emails and flyers to those who held relevant leadership roles in cancer care and ALC management and who might be interested in the study. Participants who learned about the study and wanted to participate reached out to the research team, who confirmed eligibility and offered further information about the study. Participants were informed that their identity would be protected, that their participation was voluntary and confidential, and that they could withdraw from the study without consequence at any time. Participants signed an informed consent form and completed a socio-demographic questionnaire. Participants were compensated with a gift card of their choosing, valued at $25 CAD.

### Participants

In total, 68 unique individuals participated across the two studies, which included ten patients, seven caregivers, 40 healthcare providers, and eleven healthcare decision-makers (see Table 1). In the context of this research, “healthcare decision-makers” are defined as leaders on hospital units who direct care, manage units, and/or supervise frontline staff. As noted, “caregivers” are defined as family, chosen family, or friends who provide support. Some participants had dual roles; for example, one caregiver also had experience as a patient, and three caregivers also had experience as healthcare providers. To be eligible to participate in the research, participants needed to be 18 years of age or older. For the cancer care study, participants must have received or provided cancer care services during the COVID-19 pandemic. For the ALC study, participants must have experienced, provided, or managed ALC care in hospitals during the COVID-19 pandemic.

**Table 1:** Participants across the cancer care and ALC studies.

Study	Patients	Caregivers	Healthcare providers	Healthcare decision-makers	Total per study
Cancer care study	8	5	14	5	32
ALC study	2	2	26	6	36
Total across studies	10	7	40	11	68

Overall, participants were between the ages of 30 and 69; 55 were female, seven male, and six did not say. Thirty participants self-identified as white, seven as European, two as Black, one as Japanese, one as Latin, two as Canadian, and the rest did not say; 33 were university-educated, seven college-educated, one high school-educated, two did not graduate high school, and the remainder did not say. Providers and decision-makers had up to 39 years of experience in their field and had between half a year and 28 years of experience in their current role.

### Interviews and focus groups

In total, five focus groups and 53 one-on-one, semi-structured interviews took place (45–120 min in duration). Both focus groups and interviews were offered to accommodate participants’ preferences and were used consistently across both studies. Focus groups and interviews across both research projects were audio recorded and conducted using Zoom communication software (41). Examples of questions asked during focus groups and interviews in each study are reported in Table 2.

**Table 2:** Examples of focus group and interview questions.

Participant type	Cancer care study questions	ALC study questions
Patients	Tell me about the type of care you were receiving prior to the pandemic. In what ways, if any, did your family member or friends’ assistance with your care change during the COVID-19 pandemic? What do care providers need to know about your experience that can help them improve how they provide care?	How did staff communicate with you about your care/discharge plan? Can you tell me about the discharge/transition process (the move out of hospital)?
Caregivers	In what ways (if at all) did care change during the pandemic? Were there particular things about care that did or did not work well? What (if any) delays did [patient] experience in care due to the pandemic?	In what ways, if any, was [care recipient’s] discharge plan/destination (where they were supposed to go) impacted by COVID-19? Tell me about any visitor restrictions put in place at the [hospital, long-term care facility].
Healthcare providers and decision-makers	What was your role throughout the pandemic? In what ways has the pandemic changed your clinical practices and how you provide care for patients? How did delays in care impact your team functioning?	How were ALC patients impacted during COVID-19? What [organizational] directives, policies or procedures were put in place in the [care organization] during COVID-19 in order to manage the ALC problem?

### Analysis

All audio recordings were transcribed and then transcripts were verified for accuracy in comparison to the recording. Transcripts were anonymized by eliminating identifying information. Pseudonyms were assigned. Using NVivo qualitative data analysis software (42), the authors engaged in codebook thematic analysis (43). A flexible codebook, which developed as new insights arose from the data, was established (44, 45). First, five transcripts from each study were coded independently by two researchers (AB and DJ for the cancer care study; AB and another research staff for the ALC study). The researchers who engaged in coding then met to discuss evolving codes, meaning within the data, and how each of their social locations influenced the way in which they entered into data analysis. With mostly congruent codes, AB continued to code remaining transcripts with a second analyst reviewing and adding codes. Codes with overlapping meaning were then combined and codes no longer relevant removed. Initial themes and related subthemes were developed from the codes. DJ, AB, and KK noticed an overlap in codes and related themes across the cancer care and ALC projects, specifically related to the work to manage and work around the “no visitors” policy. The authors therefore extracted the overlapping themes from both projects and continued analytic writing of the data together to convey the observed work processes independently occurring across in-hospital units.

## Findings

Throughout the pandemic, hospitals were subject to numerous province-wide, top–down standards in which there were persistent changes, reversals, and amendments as new information about COVID-19 and best-practices safety measures emerged. Hospitals received these directions often from provincial government bodies (e.g., Ontario Ministry of Health). Given the rapidly changing standards and varying contexts and circumstances within each hospital unit (and also across hospitals), inconsistent and often inequitable application of “no visitors” restrictions materialized. Participants did not mention the existence of particular top–down enforcement measures taken by the government to ensure the standards were applied. They indicated a sense of doing their best to adhere to the evolving provincial direction during an immensely challenging time. If permitted at all, when individuals wished to enter the hospital during the implementation of the “no visitors” restrictions, they had to be screened, often by a non-healthcare provider, followed by gaining entry to individual units within the building.

Overall, managing the “no visitors” policy and eventual pushback against it from various stakeholders generated a significant burden of work for patients, caregivers, healthcare providers, and healthcare decision-makers at a time when difficult emotions were at a high and resources and capacity were at a low. However, some participants did discuss the benefits of the policy to their overall workplace environment. For example, Sophia (clinical manager, cancer care study) pointed to the decrease in “*outbreaks in the hospital*” of various diseases by limiting those allowed entry. Tyler (radiation therapist, cancer care study) highlighted the additional “*research about decreasing the number of [treatment] appointments that they [patients] can come in for*” spurred on by the guidance to limit those entering into the hospital.

Participants worked to bridge the gap between patients and their caregivers by managing and working around the “no visitors” policy. Five themes are discussed below that depict this burden of work: (1) work of making individual exceptions to the “no visitors” policy, (2) work of standardizing exceptions, (3) work to remedy and navigate inconsistencies across hospital units or partner organizations, (4) workarounds to gain in-hospital entry via “hot words” and sneaking in, and (5) workarounds when in-hospital entry was not possible via technology and talking through windows. Conclusions drawn across each of the themes are based on analysis across all participant types.

### Work of making individual exceptions to the “no visitors” policy

Despite the “no visitors” policy originally being a blanket of restrictions applied to all, at times, some exceptions were made on an individual basis. In particular, individual exceptions were sometimes made if a patient became “*really sick during their [treatment]*” (Ingrid, nurse, cancer care study); if the patient was “*physically or mentally unable to handle themselves*” (Bronaugh, patient, cancer care study); if they had “*significant emotional distress*” (Janet, social worker, cancer care study), and “*if you were unsafe to be alone*” (Charlie, manager, ALC study). Charlie (manager, ALC study) detailed being “*so short staffed that we were unable to get in and do proper feeding for patients*.” This formed the basis for another individual exception: “*When we were really short staffed, and people [caregivers] were willing to come in to feed, we would allow them to come in*” (Charlie, manager, ALC study). However, Chance (radiation therapist, cancer care study) explained that these exceptions were still “*heavily screened*.”

Some patient participants pointed to individual exceptions made by their healthcare providers, which provider participants did not mention. For example, Danni (patient, cancer care study) described the lengths her healthcare provider went to after her caregiver was turned away at the hospital doors to ensure she had the support deemed necessary:

*They [provider] said, “if that kind of stuff ever happens again call me, we will meet you at emerg and help get you through.” My care team is exceptional. So, the “no visitors” policy, well, I recognize why it wasn’t consistently applied and it was hard.*

While Danni’s (patient, cancer care study) provider considered patient support critical and thus worked to advocate for intentional breaches of the rules for their particular patients, not all patients had access to this capital. When a healthcare provider did not assume an advocacy role, whether a caregiver was let into the hospital “*all depended [on] who the screener was at the door*” (Danni, patient, cancer care study). At times, screeners used their own discretion for one-time, individual exceptions. This became frustrating for providers: “*[Screeners] would say to them [caregivers] at the front door—it was frustrating, because they would say—‘oh, I’ll let you in just this one time’*” (Lex, nurse, cancer care study).

Part of this frustration was that “*it [was] kind of up to the [screener] to remember that that was a one-time [exception]*” (Lex, nurse, cancer care study). If that one-time visitor tried to gain admission again the next day, the screener might not remember the individual for whom the exception was made. That person may then receive yet another “*one-time exception*.” Whether a caregiver was let in was thus often an inequitable “*luck of the draw … if you’ll get an exemption or if they’ll [screeners] turn their back*” (Cassandra, discharge planner, cancer care study). This led patients and caregivers to engage in the often emotional work of justifying why they should qualify to be exempt from the policy. Danni (patient, cancer care study) clarified an instance in which she explained to a screener why her caregiver should be allowed in-hospital entry, which led her to feel uncomfortable and cry:

*I was going in for a biopsy. I had developed lumps in my abdomen and my father was with me. I was terrified and I was told he could not come in. And I’m explaining to this screener … crying in front of this screener at the front of [study site], at the entranceway, and it was just absolutely mortifying.*

In other instances, healthcare provider participants highlighted that individual exceptions required a lengthy work process within a particular hospital unit where it was “*the manager who had to sort of determine whether it [the requested individual exception] was sort of, within parameters*” (Charlie, manager, ALC study). This was because, often, providers did not “*have authorization to [approve one-time exceptions]*” (Ainsley, nurse, cancer care study). June (manager, ALC study) elucidated the process to obtain individual approval and justification for why this was necessary:

*There was a period of time where we had to get director approval to allow an extra visitor in for a client … [you] basically write a briefing note to say, “this is the client’s situation, this is why I’m advocating for an extra family member. It’s going to benefit their cognition, their mental health,” whatever it might be. Because the visiting rules were so strict because we were terrified of, you know, being a statistic like we have seen where COVID’s running rampant … and you have had COVID-related deaths because we did not do our due diligence to, you know, monitor things [visitors] appropriately.*

Lex (nurse, cancer care study) detailed that she could also clear individual exceptions with a social worker and that she had to indicate the exemption in the patient’s chart: “*We usually worked with social work and management like our leadership to be like ‘this patient needs a visitor, because X, Y, and Z’. And then we were able to put a flag on their chart for that*.” Social workers helped providers to “*determine … their [patient’s] mind function and … [if caregivers should be warranted] to come around*” (Lex, nurse, cancer care study). Ainsley (nurse, cancer care study) detailed that to allow an individual exception, “*you had to have the contact info and name of that person*” and when the caregiver showed up, they had to “*get checked off on the list*,” which had to “*match*” who had been individually approved.

Ainsley (nurse, cancer care study) juxtaposed the apparent simplicity of this work process against the weight of the added burden of labor:

*The big thing is human resources, so working short staffed has been something that’s been only getting worse over the years. Pandemic, I think, really is what shed light towards that… So even though … being asked, “is so-and-so on the list? Can they visit?” even though it seems so insignificant, in your already busy day with your patient care load being two extra patients, it takes a lot of that nurse to have to do those extra tasks.*

Ocean (manager, ALC study) pointed to the stress that managers experienced when they had to make difficult choices about for whom individual exceptions would be made:

*All those requests came to the manager. I’m telling you, that was pretty stressful, to tell people they could come in or not come in. Right? Like you were the gatekeeper. And I do not like being the gatekeeper of telling families they can see each other or not.*

This extra work often made healthcare providers “*feel unsupported*” as they were “*doing extra work and not getting extra help*” (Ainsley, nurse, cancer care study). Overall, making individual exceptions to the “no visitors” policy was an additional burden of labor for healthcare providers which “*took up a lot of time*” (Sam, nurse, cancer care study).

### Work of standardizing exceptions to the “no visitors” policy

Healthcare provider participants indicated that some units engaged in an additional process of work to generate standard exceptions. This was largely (1) to alleviate the burden of work from making numerous individual exceptions to the “no visitors” policy, and (2) because healthcare leaders also came to “*realize [that] there were essential reasons why people need[ed] to come in*” (Ocean, manager, ALC study). This work of standardizing exceptions happened sequentially, as a solution to the high degree of work identified to make individual exceptions, and thereby constituted a distinct work process. Cassandra (discharge planner, cancer care study) explained that her manager had “*noticed that we were always coming for [individual] exceptions*.” Her manager therefore asked her team to “*sit down and make suggestions*” for “*a processes for how we could allow [standard] exemptions*” which would provide “*guidance*” and avoid “*constantly hav[ing] to … move up the chain to get the ‘OK’*.”

Some of these standard exceptions included those with “*cognitive impairment, physical mobility issues*” (Janet, social worker, cancer care study), “*if they were delirious and they needed a friendly, familiar face*,” “*if they [caregiver] needed to feed the patient because they were not eating*” (Ocean, manager, ALC study), when the patient was “*a little younger*” (Ingrid, nurse, cancer care study), for those needing “*translation help*” (Janet, social worker, cancer care study), if “*they did not even understand what we were saying to them*” (Ocean, manager, ALC study), for “*diagnosis or bad result[s]*” (Ingrid, nurse, cancer care study), and “*if they [patient] were end of life*” (Ingrid, nurse, cancer care study). May (manager, ALC study) explained that formal exceptions could be made for some of the aforementioned reasons even during a COVID-19 outbreak:

*Of course you have got your COVID-positive patients who really fall under the outbreak category and should not have any essential care partners. But, you know what? We can allow them for patients who are confused, hard of hearing, you know, language barriers*.

However, these standard exceptions still “*w[ere]n’t a guarantee*” since caregivers “*could still get to the screeners at the [front entrance] door and be turned away*” (Janet, social worker, cancer care study).

Healthcare provider participants indicated that the additional workload unique to the process of making standard exceptions resulted when healthcare providers needed to schedule and manage designated visitor check-ins. The purpose was to control how many additional individuals would be on their unit at once. It was “*the nurse’s responsibility to write this information down*,” managed on a “*clip board beside our secretary desk*” (Ainsley, nurse, cancer care study). Sam (nurse, cancer care study) detailed: “*We could only schedule people for two hours a day. Like two hours a day, that was very difficult*.” Sandy (director, ALC study) explained how formalized exception guidelines regarding the number of caregivers and length of stay permitted on her unit varied with the rate of COVID-19 infection present:

*We came out with the different guidelines on who could visit and when. … when we are at different levels [of COVID-19 infection], how many people could come in, how—length of time and that [varies]. … At first those [standardized exceptions] were kind of really restrictive and we really loosened them a lot. And now it’s always err on the side of compassion.*

Recognizing that the initial policy was overly restrictive and obstructive to providing adequate care, the need for generating clear and compassionate rules was evident. These rules were helpful to providers in determining who met the standard exemption threshold.

One standard exception eventually made was the allowance of “*one person [formally designated visitor] that can visit [each] patient*,” termed “*ECPs—extended care partners [or essential care partners]*” (Ainsley, nurse, cancer care study). However, this meant that the entire burden of care fell directly on one person. Mave (radiation therapist, cancer care study) described,

*… you could see the toll because it was one person. … It was one specific person and had to be that same person. There was no other person allowed. So you can see the strain of someone having to come for 30 days while trying to juggle their own life, their own COVID pandemic, and everything else going on, on top of this disease.*

Being the sole caregiver was therefore a difficult responsibility to bear alone and often resulted in “strain” and burnout.

In cases when caregivers burned out, a non-designated individual often showed up as a substitute ECP. Ainsley (nurse, cancer care study) described how this “*was difficult*” because providers “*ha[d] to go through a process of swapping out [the designated caregiver] name to somebody else’s name*” if permitted. The new designated caregiver would then also need “*to be approved [by a manager] as the [new] care partner for that patient*” since it involved “*a new person entering the building*” (Ainsley, nurse, cancer care study). In response to these complexities, some providers felt that the “no visitors” policy and related formal exceptions “*sometimes caused conflict*,” and “*got a little bit murky at times*” (Ainsley, nurse, cancer care study). This often led providers to “*never know when to say yes or no*” to letting additional caregivers in-hospital (Ainsley, nurse, cancer care study).

### Work to remedy and navigate “no visitors” policy inconsistencies across hospital units and partner organizations

With formal and informal exceptions to the “no visitors” policy made at the front entrance and on particular units, there were inconsistencies with who was granted entry into the hospital. Jess (social worker, ALC study) pointed to broader inconsistencies in the “no visitors” policy in terms of how often it changed: *“[The hospital] had so many different iterations of the visitor policy … and tons of complaints and things to the manager about the visitation*.” Saturn (manager, ALC study) detailed the magnitude of the frequent changes and the additional work this generated:

*Every Friday the [Ontario] ministry [of Health] would come … there was some directive, “You’re no longer having visitors.” And it was never an easy directive. And it was never quite clear and we had to figure it out in all of six minutes. … I could not actually believe what was in front of my eyes. … The decisions were happening 24/7 and the workload became 24/7.*

Healthcare provider participants highlighted that inconsistency and lack of standardization in the exceptions made *across* in-hospital units within one institution generated further work to manage. May (manager, ALC study) explained that these inconsistencies came to fruition since “*the managers of each unit manage their own units and make [independent] decisions*.” Cassandra (discharge planner, cancer care study) described how the “*inconsistency amongst floors … caused uproar*” given the different definitions of conditions and designations across units. For instance, as Cassandra (discharge planner, cancer care study) described, the definition of “dying” differed on general medical units where patients often recovered versus palliative units where illnesses were terminal: “*On medicine floors their definition of dying is … metastatic cancer and could die in the next few months. … On our floor it’s actively dying, which looks very different*.” In response to this inconsistency, some providers worked to more consistently define what “dying” meant. This was a difficult task given that providers were already under-resourced and such definitions were context-dependent. Cassandra (discharge planner, cancer care study) continued: “*We have so many sick patients. And you advocate that can the units be more exact, and, with their definition*.”

Inconsistencies in the “no visitors” policy also extended across hospitals. For example, Jade (social worker, ALC study) explained that the nearest regional hospital eventually relaxed their “no visitors” policy so that unvaccinated caregivers could enter the facility as long as they passed a symptom-checklist screening. The local hospital at which she worked, however, was *not* allowing unvaccinated individuals entry; this ignited confusion and frustration for caregivers navigating the inconsistencies across sites and additional difficulty for providers to manage:

*When they [the regional hospital] changed their policy to open up to visitors, as long as they [caregivers] passed the rest of the screening they did not have to be vaccinated. But our [local hospital] rule had not changed. It created a lot of tension for a lot of families because they were like, “Well, I was able to visit the regional [hospital]. Now my mom’s here where she needs more support with rehab and I cannot—I’m not even allowed in the building.” And it would—yes, create a lot of frustration.*

Both patient and healthcare provider participants felt that these inconsistencies were not “*fair or equitable*” (Mani, discharge planner, cancer care study). Ingrid (nurse, cancer care study) elucidated how “*the problem is then we have a patient walking with a family member, and everyone else is wondering, ‘well, why not me?*”. Danni (patient, cancer care study) confirmed the difficulty in witnessing other caregivers present after being told she was not permitted support: “*One time I did ask the primary nurse if my fiancé could be with me and she had said that, ‘no, no visitors allowed’. But I had just seen screeners let other visitors in!*” (Danni, patient, cancer care study). In response to these inconsistencies, caregivers would often sacrifice the most expert care for a unit with more lenient restrictions. Cassandra (discharge planner, cancer care study) described how the unit where she worked had strict “no visitors” policy enforcement. Despite being better equipped to care for palliative patients, caregivers were upset when the individual they were caring for was moved to her unit: “*Families were then mad that they had to come down here [specialized unit], although, arguably, our team is the better team to provide their medical care because of our training*.”

Mani (discharge planner, cancer care study) continued to explain that patients and caregivers pushed back against the inconsistency and providers had to work to reiterate their unit’s rules. This meant that providers bore the heavy blame for a policy they did not create but were required to enact:

*I heard many times, “Isn’t your role to advocate? … can’t you make an exception? This is my situation.” … As much as you would say, “I do not have the power to change that.” They still saw that as, “I think you could do something if you really wanted to.” And so, that took a toll on them. And sometimes being asked to be the ones to deliver that message, to just say to families, “no you cannot come in” … It just was hard.*

Events such as these left some providers feeling unsupported, wishing they had been more prepared to undertake these difficult conversations while required to enforce the sometimes-controversial rules. Ocean (manager, ALC study) described:

*There’s a lot of inconsistencies. … And you did not really give us a lot of training on what to say. And what’s our backup when the [caregivers] are like, “no way, I’m not leaving”? … So you are like how much do I need to … keep pushing when I know that if they keep pushing back, they are not—they are going to get whatever they ask for. So, I think having a consistent message and giving the managers who have to give these messages a little bit more training and support to say no, this is really what we want.*

### Workarounds to gain in-hospital entry via “hot words” and sneaking in

Despite the significant work put into making individual and standard exceptions to the “no visitors” policy and working to remedy inconsistencies, some caregivers were still unable to gain entry in-hospital. Caregivers thus often observed and learned of other workarounds to support in-person visits. May (manager, ALC study) said, “*there’s ways around all the rules*.” Of note were informal “hot words,” which would often grant automatic entry into the hospital. These included, “*translation, mobility, power of attorney*,” “*neurological deficit*,” and “*significant emotional distress, cognitive impairment*.” Mave (radiation therapist, cancer care study) explained that “*some people knew those words*” and that “*staff members kind of let it slide*” when used: “*We just kind of knew those were the hot words that if you say it, you are good*.” “Hot words” were thus not an individual exception based on a unique circumstance, nor a formal exception approved in advance, but rather an informal workaround that could apply to a large subset of individuals, requiring no more justification than the utterance of the words themselves.

Caregivers quickly caught on to these “hot words” and used them to circumvent the rules, regardless of whether it was true to their experience. Jess (social worker, ALC study) explained that despite the “*screeners at the door*,” “*people would lie*” and then “*sneak in*,” which was “*a really difficult thing to monitor*.” Lex (nurse, cancer care study) clarified that “*there was no way at the front door for that to be corroborated*,” and as Jules (caregiver/clinical educator, cancer care study) described, once inside, “*typically, they are [providers] not going to tell you to leave*,” even if the providers knew that the “hot words” did not truthfully apply to a particular patient caregiver duo. Another workaround caregivers found was saying to screeners at the front door that they were a patient, since this was not easily corroborated on the spot: “*Family members … started to figure out— ‘oh I’ll just say that I’m a patient and I have an appointment’*” (Lex, nurse, cancer care study).

Other times, providers had to contend with family members sneaking into the hospital without the use of “hot words.” Sam (nurse, cancer care study) described,

*We would have family members sometimes sneak in, hide in the bathroom. Nursing would see them, but like would not say anything to avoid the confrontation. … It was hard to have that conversation. To say “I’m sorry, you have to leave your loved one. You cannot be here.”*

Providers needed to work to discuss amongst one another whether action would be taken on those who snuck in, and if not, then were put in the uncomfortable position of allowing a breach of the rules. Caregivers, like Jules (caregiver/clinical educator, cancer care study), admitted to trying to “*push the rules*” and “*snuck in lots of times when I really wasn’t supposed to be there*.”

### Workarounds when in-hospital entry was not possible: technology and talking through windows

When caregivers were unable to gain in-hospital entry, many tried to be there for loved ones using technology such as video conferencing with the use of a tablet. This created more work for providers to facilitate, which often involved scheduling iPad use, arranging a time to connect, ensuring families had adequate equipment, and dealing with the difficulty of using the technology itself. West (nurse, cancer care study) described,

*On the in-patient setting … we had like two iPads that we used, and we could do video, arrange video chats for the patients. But the issue with that was technology. Sometimes if the family member at home does not have the right app, they could not connect.*

June (manager, ALC study) specified that the group of older adults with whom she worked “*is not really a computer-savvy population*.” Jade (social worker, ALC study) clarified the difficulty of connecting older adult family members who struggled to use the technology (e.g., audio and video communication):

*During the peak of things and when we were having outbreaks then there was tele-visitation. … And when you are working with the geriatric population and you have a client who’s maybe 95 years old, their kids are in their 70s. … With our clientele … sometimes they would be like, “I cannot see it.” Or they … could not quite hear it. … It’s not like our clientele could be independent with it.*

Therefore, despite providers engaging in extra work to facilitate caregiver involvement using technology, unstable connections made it difficult to obtain care-related information, and caregivers found it difficult to participate in patient care “*navigat[ion] from afar*” (Jules, caregiver/clinical educator, cancer care study).

Healthcare provider participants like June (manager, ALC study) continued to explain that often, “*staff just did not have time … to sit there and hold an iPad*.” This meant that if facilitation of tele-visitation “*relied on other staff members it definitely was not getting done*.” West (nurse, cancer care study) also described difficulty finding personnel to facilitate the interaction: “*And finding somebody to arrange it. It was usually always left to the bedside nurse or the charge nurse if they had time. … So, it was like a human resource issue*.” Jade (social worker, ALC study) expanded that upon recognition that healthcare providers were unable to take on the work of facilitating tele-visitation, an extra individual was hired to help: “*Their whole job was tele-visitations. … You book a time and then that staff member would coordinate that [tele] visit*.” However, she continued to explain workflow challenges with bringing a new staff member onto an over-capacity unit, which still required support from healthcare providers:

*The challenge with that is now you have an outside person coming in who is—well, then maybe does not know the client so it’s maybe not as easy, right? … You’re going to have to coordinate to bring the client out of the room … to have that visit so they can actually maybe hear without having tons of background noise. But then that created a whole other obstacle of like that means the client has got to get a nurse to transfer them up, get them out of there, get them down to another room. … It just led to lots of other—even the things that you think would be simple would become complicated.*

June (manager, ALC study) continued to explain that healthcare providers “*had all this extra work and now we have to try and facilitate visits electronically or via phone-call. It was really, really, really challenging*.”

While most healthcare provider participants indicated that technology often generated an additional burden of work, other healthcare provider participants like Mikah (oncologist, cancer care study) pointed to some benefits: “*[With technology] we bypassed all of that [registering, waiting] by just, like, phoning [patients] … it gives us more time with the patients … They can have their people [caregivers] with them*.” Meeting via phone was beneficial in reducing waiting room times and allowing patients to have in-person caregiver support amidst in-hospital “no visitors” restrictions. Ingrid (nurse, cancer care study) described virtual appointments as “*positive for people who were able to use [technology]*.” However, she also pointed to the difficulty when “*technology would just fail*.” Cassandra (discharge planner, cancer care study) echoed the challenge that “*not everybody had access to Zoom or FaceTime*.” Despite the benefits of technology to accommodate patient support, healthcare provider and patient participants clarified that virtual support should “*not take the place of in-person*” (Mani, discharge planner, cancer care study). Danni (patient, cancer care study) also explained how connecting with caregivers via video was insufficient compared to in-person support: “*I was told, ‘if you really need support just put them on Facetime’ … and I kind of thought, it’s not the same thing*.”

Caregivers who were unsuccessful in entering the hospital and had loved ones on the first floor often sat by the window of their room. Cassandra (discharge planner, cancer care study) described,

*We’re on the main floor, like ground level, so at least families could look through a window … for hours they’d sit. … And then the patient would have their cell phone on, their loved ones would have their cell phone on, the nurse’s sometimes will be able to move the bed … so they could see the window as well. People wrote love notes and taped them on the windows. … That was a nice thing that we could do that other floors could not. That was a bit better than a FaceTime or Zoom.*

Saturn (manager, ALC study) expanded that for those who did not have a room with a window, healthcare providers took on the extra work of bringing the patient to a hallway window to connect with loved ones.

*We were lucky because we had windows on the bottom floor so the clients would come to the windows and the family members could visit them from the windows. But then we had to actually get staff who could take people downstairs and spend time at the windows, which was another thing we had to organize.*

She continued to explain that during temperate weather conditions, healthcare providers facilitated outdoor visits:

*We had outdoor visiting for awhile. So we had to actually pay for a large tent because you have to think about weather and we were allowed to have people see their loved ones outside and we had to arrange again staff to come and take them outside and to do that.*

Caregivers often went to creative lengths to support their loved ones in the best way they could, which required work from both themselves and providers to facilitate. Sitting by a window to be with a loved one was preferred over technology such as FaceTime, demonstrating the basic need for the physical presence of loved ones. While sitting at the window was preferable to a virtual call, Mani (discharge planner, cancer care study) highlighted that “*it’s no comparison to being able to be in a room and hold your mom’s hand. To be at a window is very, you just cannot compare that*”. See below for a summary of findings (see Table 3).

**Table 3:** Summary of findings with extended quotes.

Theme	Key Points	Quotes
Work of making individual exceptions to the “no visitors” policy	Individual exceptions included for: physical or mental concerns, emotional distress, for feeding concerns, if treatment made the patient sick.	Individual exceptions were sometimes made if a patient became “really sick during their [treatment]” (Ingrid, nurse, cancer care study); if the patient was “physically or mentally unable to handle themselves” (Bronaugh, patient, cancer care study); if they had “significant emotional distress” (Janet, social worker, cancer care study), and “if you were unsafe to be alone” (Charlie, manager, ALC study). “When we were really short staffed, and people [caregivers] were willing to come in to feed, we would allow them to come in.”—Charlie (manager, ALC study)
	There were inconsistencies in individual exceptions made.	“They [provider] said, “if that kind of stuff ever happens again call me, we will meet you at emerg and help get you through.” My care team is exceptional. So, the “no visitors” policy, well, I recognize why it wasn’t consistently applied and it was hard.”—Danni (patient, cancer care study) “[Screeners] would say to them [caregivers] at the front door*—*it was frustrating, because they would say*—*‘oh, I’ll let you in just this one time’.” (Lex, nurse, cancer care study) “It [was] kind of up to the [screener] to remember that that was a one-time [exception].” (Lex, nurse, cancer care study) “Depending on the [screener], they’d let people in.” (Cassandra, discharge planner, cancer care study) “[It was a] luck of the draw … if you’ll get an exemption or if they’ll [screeners] turn their back.” (Cassandra, discharge planner, cancer care study)
	Some patients worked to justify why a caregiver should permitted to provide them individually with support.	“I was going in for a biopsy. I had developed lumps in my abdomen and my father was with me. I was terrified and I was told he could not come in. And I’m explaining to this screener … crying in front of this screener at the front of [study site], at the entranceway, and it was just absolutely mortifying.”—Danni (patient, cancer care study)
	Individual exceptions could be cleared with a manager or a social worker.	“Everything would have to go through the manager.” — (Sam, nurse, cancer care study) “[Providers did not] have authorization to [approve one-time exceptions].” — (Ainsley, nurse, cancer care study) “[It was] the manager who had to sort of determine whether it [the requested individual exception] was sort of, within parameters.”— Charlie (manager, ALC study) “We usually worked with social work and management like our leadership to be like ‘this patient needs a visitor, because X, Y, and Z’. And then we were able to put a flag on their chart for that.”— Lex (nurse, cancer care study)
	Sometimes, there was a lengthy work process within hospital units to make individual exceptions.	“There was a period of time where we had to get director approval to allow an extra visitor in for a client … [you] basically write a briefing note to say, “this is the client’s situation, this is why I’m advocating for an extra family member. It’s going to benefit their cognition, their mental health,” whatever it might be. Because the visiting rules were so strict because we were terrified of, you know, being a statistic like we have seen where COVID’s running rampant … and you have had COVID-related deaths because we did not do our due diligence to, you know, monitor things [visitors] appropriately.”—June (manager, ALC study)
	Individual exceptions were a significant source of labor for healthcare providers given the lacking capacity and resources.	“The big thing is human resources, so working short staffed has been something that’s been only getting worse over the years. Pandemic, I think, really is what shed light toward that. So working short staffed with more responsibilities. So even though … being asked, “is so-and-so on the list? Can they visit?,” even though it seems so insignificant, in your already busy day with your patient care load being two extra patients, it takes a lot of that nurse to have to do those extra tasks.”—Ainsley (nurse, cancer care study)
	Determining for whom an individual exception would be made was stressful for healthcare providers.	“All those requests came to the manager. I’m telling you, that was pretty stressful, to tell people they could come in or not come in. Right? Like you were the gatekeeper. And I do not like being the gatekeeper of telling families they can see each other or not.”—Ocean (manager, ALC study)
Work of standardizing exceptions to the “no visitors” policy	Standardized exceptions were made for those who had: cognitive impairment, mobility concerns, mental health concerns, feeding concerns, language barriers, bad results or were at the end of their life.	Standard exceptions were made for those with “cognitive impairment, physical mobility issues” (Janet, social worker, cancer care study), “if they were delirious,” “if they [caregiver] needed to feed the patient” (Ocean, manager, ALC study), when the patient was “a little younger” (Ingrid, nurse, cancer care study), “for patients with … language barriers” (Chance, radiation therapist, cancer care study), if “they did not even understand what we were saying to them” (Ocean, manager, ALC study), “if people just could not cope” (Lex, nurse, cancer care study), for “diagnosis or bad result[s]” (Ingrid, nurse, cancer care study), and “if they were end of life” (Ingrid, nurse, cancer care study).
	Additional work processes were generated to allow for standardized exceptions.	“[Leaders came] to “realize [that] there were essential reasons why people need[ed] to come in”—Ocean (manager, ALC study) Cassandra (discharge planner, cancer care study) explained that her manager had “noticed that we were always coming for [individual] exceptions” and so asked her team to “sit down and make suggestions” for “a processes for how we could allow [standard] exemptions” which would provide “guidance” and avoid “constantly hav[ing] to … move up the chain to get the ‘OK’.”
	Part of the additional work was scheduling.	“We could only schedule people for 2 hours a day. Like 2 hours a day, that was very difficult.”—Sam (nurse, cancer care study)
	Standard exceptions changed according to the level of COVID-19 present.	“We came out with the different guidelines on who could visit and when. … The hospital right now is still in level red. And when we are at different levels [of COVID-19 infection], how many people could come in, how—length of time and that [varies]. … At first those [standardized exceptions] were kind of really restrictive and we really loosened them a lot. And now it’s always err on the side of compassion.”— Sandy (director, ALC study)
	The allowance of one extended care partner meant that the entire burden of care fell on one person, which led to caregiver burnout and resultantly, changes in partners.	“You could see the toll because it was one person. … It was one specific person and had to be that same person. There was no other person allowed. So you can see the strain of someone having to come for 30 days while trying to juggle their own life, their own COVID pandemic, and everything else going on, on top of this disease.”— Mave (radiation therapist, cancer care study) “[Providers] ha[d] to go through a process of swapping out [the designated caregiver] name to somebody else’s name.”—Ainsley (nurse, cancer care study)
Work to remedy and navigate “no visitors” policy inconsistencies across hospital units and partner organizations	Inconsistencies in individual and standard exceptions was difficult for all involved.	“The managers of each unit manage their own units and make [independent] decisions.”—May (manager, ALC study) “[The] inconsistency amongst floors … caused uproar.”—Cassandra (discharge planner, cancer care study) “[Some] units were doing things differently. So, people would come to our floor and say, ‘well, on this [other] unit that my loved one was just at, we actually were allowed to have … visitors’.”—Mani (discharge planner, cancer care study) “The problem is then we have a patient walking with a family member, and everyone else is wondering, ‘well, why not me?’.”—Ingrid (nurse, cancer care study) “One time I did ask the primary nurse if my fiancé could be with me and she had said that, ‘no, no visitors allowed’. But I had just seen screeners let other visitors in!”—Danni, patient, cancer care study)
	Frequently changing regulations posed challenges to equitable enforcement.	“[The hospital] had so many different iterations of the visitor policy … and tons of complaints and things to the manager about the visitation.”—Jess (social worker, ALC study) “Every Friday the ministry [Ontario Ministry of Health] would come. It was always Fridays at 4:30, there was some directive, “You’re no longer having visitors.” And it was never an easy directive. And it was never quite clear and we had to figure it out in all of 6 min. … I could not actually believe what was in front of my eyes. … The decisions were happening 24/7 and the workload became 24/7.”—Saturn (manager, ALC study)
	Part of the inconsistency was different mobilization of medical terms such as “dying” across units in the same hospital.	“On medicine floors their definition of dying is … metastatic cancer and could die in the next few months. … On our floor it’s actively dying, which looks very different.”—Cassandra (discharge planner, cancer care study) “You’re watching that day in and day out because we have so many sick patients. And you advocate that can the units be more exact, and, with their definition.” —Cassandra (discharge planner, cancer care study) “We had the exemption of less than 2 months or less than 3 months [to live].”—Sam (nurse, cancer care study)

	There was also inconsistency in application of the policy across near-by institutions.	“I know lots of times we did as well try to match what the regional hospital was doing because otherwise it would lead to a lot of difficulty. … So, when they [the regional hospital] changed their policy to open up to visitors, as long as they [caregivers] passed the rest of the screening they did not have to be vaccinated. But our [local hospital] rule had not changed. It created a lot of tension for a lot of families because they were like, ‘Well, I was able to visit the regional [hospital]. Now my mom’s here where she needs more support with rehab and I cannot*—*I’m not even allowed in the building’. And it would*—*yes, create a lot of frustration.”—Jade (social worker, ALC study)
	Less expert care was often preferred in exchange for more lenient restrictions.	“Families were then mad that they had to come down here [specialized unit], although, arguably, our team is the better team to provide their medical care because of our training.”— Cassandra (discharge planner, cancer care study)
Healthcare providers had to reiterate the rules when caregivers believed more could be done to facilitate exceptions.	“I heard many times, “Isn’t your role to advocate? … can’t you make an exception? This is my situation.” … As much as you would say, “I do not have the power to change that.” They still saw that as, “I think you could do something if you really wanted to.” And so, that took a toll on them. And sometimes being asked to be the ones to deliver that message, to just say to families, “no you cannot come in” … It just was hard.”—Mani (discharge planner, cancer care study)
Workarounds to gain in-hospital entry via “hot words” and sneaking in	Some caregivers gained entry into the hospital using hot words.	“We just kind of knew those were the hot words that if you say it, you are good.”—Mave (radiation therapist, cancer care study) These included, “translation, mobility, power of attorney,” “neurological deficit,” “significant emotional distress, cognitive impairment.” “There’s ways around all the rules. Right? You can make a lot of things fit.”—May (manager, ALC study) “There was no way at the front door for that to be corroborated, right? So patients found their own work arounds, sometimes*—*they were smart.”—Lex (nurse, cancer care study) “[I] snuck in lots of times when I really wasn’t supposed to be there.”—Jules (caregiver/clinical educator, cancer care study)
	Some caregivers lied to gain in-hospital entry.	“Family members … started to figure out— ‘oh I’ll just say that I’m a patient and I have an appointment’.” (Lex, nurse, cancer care study) “That was one thing. [The hospital was] locked as in [there were] screeners at the door. But still, people would sneak in. People would lie. … It was a challenge … having to tell people to leave. … That was a really difficult thing to monitor.”—Jess (social worker, ALC study)
	Once inside, caregivers were not often told to leave.	“[Once inside] typically, they are [providers] not going to tell you to leave.”— Jules (caregiver/clinical educator, cancer -care study) “We would have family members sometimes sneak in, hide in the bathroom. Nursing would see them, but like would not say anything to avoid the confrontation. You would kind of say “Hey, I think there’s a family member here in the bathroom.” And nobody wanted to say anything. It was hard to have that conversation. To say “I’m sorry, you have to leave your loved one. You cannot be here.”—Sam (nurse, cancer care study)
	Providers felt unprepared to address when caregivers were present when they should not be.	“This visitor policy, patients come to us and families come to us, and some will say, “oh, the manager just let me visit.” Or, “no, the manager stayed firm and did not let …” So, there’s a lot of inconsistencies. … And you did not really give us a lot of training on what to say. And what’s our backup when the [caregivers] are like, “no way, I’m not leaving”? … So you are like how much do I need to…keep pushing when I know that if they keep pushing back, they are not*—*they are going to get whatever they ask for. So, I think having a consistent message and giving the managers who have to give these messages a little bit more training and support to say no, this is really what we want.”—Ocean (manager, ALC study)
Work arounds when in-hospital entry was not possible: Technology and talking through windows	Technology at times facilitated patient–caregiver togetherness.	“[With technology] we bypassed all of that [registering, waiting,] by just, like, phoning [patients] … it gives us more time with the patients … They can have their people [caregivers] with them.”— Mikah (oncologist, cancer care study)

	Technological literacy and access was a barrier to connecting patients with families remotely.	“On the in-patient setting … we had like two iPads that we used, and we could do video, arrange video chats for the patients. But the issue with that was technology. Sometimes if the family member at home does not have the right app, they could not connect.” — West (nurse, cancer care study) “[The older adult population I work with] is not really a computer-savvy population.”—June (manager, ALC study) “During the peak of things and when we were having outbreaks then there was tele-visitation. So like the iPad. … And when you are working with the geriatric population and you have a client who’s maybe 95 years old, their kids are in their 70s. … With our clientele … sometimes they would be like, “I cannot see it.” Or they … could not quite hear it. … It’s not like our clientele could be independent with it.” Jade (social worker, ALC study) “Not everybody had access to Zoom or FaceTime.”—Cassandra (discharge planner, cancer care study)
	Healthcare provider capacity was a barrier to connecting patients with families remotely.	“Staff just did not have time … to sit there and hold an iPad.”— Jade (social worker, ALC study) “[If tele-visitation] relied on other staff members it definitely was not getting done.” — Jade (social worker, ALC study) “And finding somebody to arrange it. It was usually always left to the bedside nurse or the charge nurse if they had time to go get the iPad, connect to the family, take it to the patient. So, it was like a human resource issue just having somebody to get the iPad and connect it with the family member and bring it to the patient.” — West (nurse, cancer care study) “[Healthcare providers] had all this extra work and now we have to try and facilitate visits electronically or via phone-call. It was really, really, really challenging.”— June (manager, ALC study)
	Some units had additional personnel to facilitate remote connection, which had its own challenges.	“Their whole job was tele-visitations. Do these like little iPad, allow video conferencing visits with family. … You book a time and then that staff member would coordinate that [tele] visit.”— Jade (social worker, ALC study) “The challenge with that is now you have an outside person coming in who is*—*well, then maybe does not know the client so it’s maybe not as easy, right? … You’re going to have to coordinate to bring the client out of the room … to have that visit so they can actually maybe hear without having tons of background noise. But then that created a whole other obstacle of like that means the client has got to get a nurse to transfer them up, get them out of there, get them down to another room. Are they allowed out of the room or are they on isolation? … It just led to lots of other*—*even the things that you think would be simple would become complicated.” — Jade (social worker, ALC study)
	Caregivers at times sat by the window of patients’ rooms.	“We’re on the main floor, like ground level, so at least families could look through a window … for hours they’d sit, especially in the summer. … Some people just left their camp chairs next to their loved ones’ window, and then would just come every day and sit. And then the patient would have their cell phone on, their loved ones would have their cell phone on, the nurse’s sometimes will be able to move the bed … so they could see the window as well. People wrote love notes and taped them on the windows. So, for those folks who had a window, or were in a private room, that was a nice thing that we could do that other floors could not. That was a bit better than a FaceTime or Zoom.”— Cassandra (discharge planner, cancer care study)
	For patients who did not have a window, some healthcare providers brought the patients to a hallway window or outside.	“We were lucky because we had windows on the bottom floor so the clients would come to the windows and the family members could visit them from the windows. But then we had to actually get staff who could take people downstairs and spend time at the windows, which was another thing we had to organize.”— Saturn (manager, ALC study) “We had outdoor visiting for awhile. So we had to actually pay for a large tent because you have to think about weather and we were allowed to have people see their loved ones outside and we had to arrange again staff to come and take them outside and to do that.” — Saturn (manager, ALC study)
Remote connection and connection through windows was viewed as insufficient.	“I was told, ‘if you really need support just put them on Facetime’ … and I kind of thought, it’s not the same thing.”—Danni (patient, cancer care study) “We did try to video, like we did try to conference call me in. And again, the physicians and the nurses were very, very patient because sometimes it was difficult to hear, to interpret what they were saying or things like that. So, they were very patient and repeated the information that I needed and were receptive of me asking questions … the majority of the part was just navigating the system and helping her navigate, which I think … was even heightened because of the pandemic.”—Jules (caregiver/clinical educator, cancer care study) “It’s no comparison to being able to be in a room and hold your mom’s hand. To be at a window is very, you just cannot compare that.”— Mani (discharge planner, cancer care study)

## Discussion

The current study highlights the burden of work generated for all involved to devise, implement, manage, and work around the “no visitors” policy—this was a central challenge of the restrictions implemented during the COVID-19 pandemic. While the current research draws attention to the particular work generated to manage the policy, there is room for further work to better understand the historical contextualization of the restrictions, the specific timeline of their implementation, and how they changed over time as more information about COVID-19 was gleaned. However, the findings of this study revealed that organizations’ discretion in implementing government-mandated restrictions relied on the interpretation of managers and front-line teams. As a result, there were inconsistencies in implementation. This phenomenon is consistent with the theory of street-level bureaucracy [i.e., “how to treat all citizens alike in their claims on government, and how at the same time to be responsive to the individual case when appropriate” (46, p. xii)]. The “no visitors” policy therefore created a chasm between healthcare providers, patients, and their caregivers in the name of safety and generated a substantial burden of work to manage and work around.

In the context of cancer and ALC patient care, some healthcare providers and decision-makers worked to advocate for patients to allow for intentional amendments to the rules. These amendments occurred when they did not necessarily agree with the policy, were desperate for the additional assistance in care provided by caregivers, or when they more heavily weighted the importance of families being together. However, this social capital was not equitably available for all patients and led to harmful emotional strain for those who observed the allowance of support for some patient–caregiver dyads but did not receive such accommodations themselves. Inequitable enforcement of the “no visitors” policy within and across hospitals also led to strain for healthcare providers adhering to the restrictions, who then bore the blame for the rules, receiving pushback from patients and families. Learning of the individual concessions made, patients and caregivers engaged in emotionally cumbersome work to justify to screeners why they, too, should be considered as exceptions to the restrictions. This high burden of work elicited by the inconsistent exceptions made across hospital units led to further efforts to formalize standard exceptions and streamline the decision-making and enforcement process.

However, some caregivers were still denied entrance into the hospital and found workarounds to either gain entry or connect with their loved ones in other ways. While some caregivers gained knowledge of the “hot words” that would grant entry into the hospital, others snuck in. Those who did not have access to this understanding would have been substantially disadvantaged (e.g., linguistically diverse individuals, those who did not feel comfortable or have the capacity to advocate considering cultural norms, past poor experiences, and fear of being reprimanded). Such epistemic injustice is partly shaped by the lack of avenues for people to speak up about their care in ways that align with their cultural and personal values. Overall, the “no visitors” policy generated extra work (and related harms) for patients, caregivers, healthcare providers, and healthcare decision-makers to manage, make exceptions, find workarounds, advocate, and do what was necessary to connect patients with their support system at a minimum—a tenet considered vital to person- and family-centered care.

### The work of creating individual and standardized exceptions to restrictive policy

Despite previous post-hoc literature depicting the need for less restrictive “no visitors” regulations during the COVID-19 pandemic (13), limited research exists on the work of managing and working around the policy itself. Individual exceptions to the “no visitors” policy reported in the current study align with those described in other research [e.g., for younger patients, those with disabilities and behavioral concerns, end-of-life (47)]. Some exceptions reported in the literature, which were not reported in the current study, include for those in the emergency department, rehabilitation, and outpatient clinics (47). Despite participants’ descriptions of healthcare providers having minimal capacity to engage in this extra work, added processes to instate and manage these individual exceptions included: keeping up with frequently changing regulations, managing inequities related to exceptions made, and advocating for patients at the hospital entrance—all of which caused distress for multiple stakeholders. Other additional work processes included justifying and seeking approval of exceptions from managers, managing checklists and admission of individually approved “visitors,” and contending with those for whom exceptions were not made.

While healthcare provider participants were tasked with implementing the restrictions, they also reported conflicting feelings about the policy’s benefits (48) and often felt it went against their moral compass and patient- and family-centered care (22). A complex rift was therefore created between patients/caregivers and healthcare providers. This rift has been noted to have intensified into confrontational behavior from patients and caregivers who worked to stand by loved ones (especially at the end of their lives) and challenged healthcare providers tasked with applying the rules (12, 22). Concordantly, the inconsistent implementation of the “no visitors” policy has been found to have decreased patients’ and caregivers’ trust in the healthcare system (49) and thus made it more difficult for healthcare providers to build institutional and interpersonal trust with patients and caregivers, resulting in strained patient/caregiver-provider relationships and undermining the importance of caregiver presence for better health and healthcare experiences (50).

In addition to individual exceptions, there was also a novel, extra burden of work to make standardized exceptions to the “no visitors” policy (e.g., generating an approved list of common exceptions, scheduling “visitors”). One eventual standard exception was allowing *one* “essential care partner” per patient, which was another source of work for healthcare providers to manage and generated harm (e.g., burnout) for caregivers who had to bear the full responsibility of caregiving alone amidst their own pandemic experience, job, family, and other accountabilities. To avoid this extra work in a low-capacity circumstance and related harms, Kemp (51) argues for the importance of reconceptualizing the classification of caregivers, so that they are recognized as “essential care partners” from the outset of policy development, moving beyond the terminology of “visitor.” Meiers et al. (30) similarly recommend family members be reconceptualized as caregivers and receivers rather than “visitors” to support ongoing efforts to formalize caregiving roles in patient- and family-centered care. The language of “essential care partners” has already been adopted (52–54) and better represents caregivers’ vital contributions to care. Ideally, this classification will in the future implicitly prompt decision- and policy-makers tasked with the responsibility of implementing restrictive policy in response to needed increased safety measures to better value caregivers as essential to care.

### The work of supporting technological solutions to work around restrictive policy

One work-around to the “no visitors” policy reported in the current study, which further generated more work for all involved, was connecting patients with loved ones using technology-facilitated visiting (e.g., telephone, video calls). Thomas et al. (55) created a flexible, automated, and less cumbersome system for supporting sustainable virtual visiting, which accommodates different cultural and family dynamics, utilizes a private, secure, easy-to-use video conferencing platform, and does not substantially impact healthcare providers’ workload. Others, like Clarke et al. (56), draw attention to new ethical considerations for virtual visits; for example, the need for enhanced verbal articulation of emotionally charged concepts like compassion and management of emotional distress from remote connection with families (considering the loss of non-verbal communication like body language and touch). More creative methods to connect patients with loved ones in the current study included sitting by a patient’s in-hospital window while simultaneously communicating via telephone, a finding also demonstrated in the long-term care facility context (57). However, similar to the current study, participants in other research also report that the use of these alternative methods of connection is “not the same as being there [in-person]” (57, p. 23).

### The work of managing the consequences of inconsistencies in restrictive policy

One challenge central to the work of making individual and standardized exceptions to the “no visitors” policy was applying such allowances consistently and equitably (58, 59). The difficulty in carrying out this task led to consequences that required further work to manage. A trade-off, therefore, exists when it comes to the differential application of policy—adaptability allows for flexible consideration of individual context and varying circumstances, while at the same time it may lead to inequity and variability in its implementation. In particular, inconsistencies arose when different screeners drew disparate conclusions about for whom an exception may be made, when some providers engaged in more advocacy work than others to allow specific patients to have in-person caregiver support, and when units across the hospital had different exception rules. Other studies, too, have reported inconsistencies in the “no visitors” policy across different hospitals, generating difficulty in the equitable and ethical enforcement of the rules (60), especially for those traversing multiple units or institutions (47). Interestingly, participants in the current study expressed a preference for patients to be on units with more lenient “no visitors” restrictions, even if it meant being on a unit with less expertise to care for a patient’s medical condition, demonstrating the importance of including caregivers on healthcare teams and within the creation and implementation of relevant policy.

Another inconsistency in the application of the “no visitors” policy was different units’ varying mobilization of certain terms. For example, in the current study, participants described that an exception to the “no visitors” policy was if a patient was “dying”; however, the definition of “dying” differed across units. Participants did not point to legal or statutory definitions of the term; however, this may be a pertinent avenue for future research to better understand the ways in which this type of guidance is (or is not) implemented across (and within) Ontario hospitals. Inconsistent definitions and communication regarding death and dying, in addition to uncertainties and inequities around the execution of end-of-life planning, led to what others have termed “death unpreparedness,” defined as when “the processes of information, communication, and relationship between the dying person, family, and HCP [healthcare providers] are compromised” (61, p. 11). These inconsistencies were a source of stress and emotional strain on patients and caregivers who were barred from providing in-person support, which further created more work for healthcare providers to manage grievances raised by caregivers who were frustrated by their allowance to support loved ones on particular floors but not others. In other literature, the “no visitors” restrictions were deemed “particularly vile” given the lack of consideration of “the individual and social costs associated with dying alone” for all involved; recommendations have therefore been made to “democratize” visitation policies by using a “patient rights framework” (62, p. 1).

Overall, these inconsistencies and the consequent difficulty for patients, caregivers, healthcare providers, and decision-makers are symptoms of a system (across hospitals and within hospitals across units) that is not integrated. To address the inequities inherent in the inconsistent application of the “no visitors” policy and related exceptions made, Olszewski et al. (59) and their broader team (including stakeholders) put forth a tool to “apply an equity framework to the COVID-19 visitor restriction policy” (p. e419) with strategies including: assembling diverse stakeholders, making records of noted inequities, working to understand the underlying causes of the inequities, and weighing the inadvertent outcomes of and alternatives to such a policy. These strategies, in addition to consultation with various stakeholders (especially those with lived experience), may also be considered by health and social care policymakers during future health crises when considering “no visitor” restrictions. Honarmand and Mehta (63) too make recommendations relevant to policymakers surrounding clearly defining exceptions to such policies to curtail inequitable implementation.

Of note are select health systems in Canada, including Kingston General Health Sciences Centre and Saskatchewan Health Authority (64), which applied these measures by including patient partners in the implementation of the “no visitors” policy during the pandemic. These examples demonstrate that the sentiment of caregivers as “essential” to healthcare system operations (20, 21) must be put into action—caregivers are not truly considered essential unless they are included in care and related policies that affect their participation in the healthcare system.

### Striking the balance between including caregivers as members of the healthcare team without misusing or expecting unpaid work

Caregivers are vital members of the healthcare team, without whom patient safety, patient- and family-centered care, and quality of care decrease (22). Ideally, the healthcare system would have the capacity and resources to support the entirety of care (65) without having “dangerous gaps,” which caregivers fill on an uncompensated basis, constituting a “shadow workforce” (66, p. 1005). It is vital to ensure healthcare resources for when caregivers are unable to engage in this role (e.g., due to systemic and social circumstances such as inability to reduce work hours, lack of finances to support extra caregiving work, etc.). Having better baseline system preparedness to provide resources when caregivers are unable to engage may also act as a protective measure to ensure that those who do participate in caregiving work are not taken advantage of.

To better support caregivers who are able and wish to contribute to healthcare teams, Leykum et al. (67) recommend the provision of “formal recognition,” integration of caregivers into healthcare strategies, creation of explicit caregiver support policies, provision of enhanced care coordination personnel, financial support for transportation, improved respite, and access to skills training (p. 1266). Kuluski et al. (68) additionally propose 12 principles to support caregiver inclusion within the healthcare system, some of which include improving policy infrastructure (e.g., incentives for institutions and individual providers to engage caregivers), making structural amendments (e.g., physical space for caregivers during care interactions), and engaging caregivers at an early stage (e.g., prior to the diagnosis of a health issue).

### Applying lessons learned from previous pandemics to future health crises

Reflections on lessons learned from previous pandemics predating COVID-19 have centered on addressing healthcare worker safety (34), mitigating the spread of disease (69), and the need for better public health and in-hospital preparedness (33). The minimal consideration of the consequences of “no visitors” restrictions, in conjunction with the knowledge gained from the COVID-19 pandemic regarding this topic, demonstrates the importance of learning from the impact of this policy and applying lessons to future emergency crises to inform policies and processes that take the needs and preferences of patients and families into account.

These lessons learned, therefore, have further implications for health and social care policymakers. Other work has highlighted key considerations that policymakers may consider when implementing visitor restrictions during health crises. For example, McDougall et al. (32) “argue that an ethically justified hospital visitor restriction policy has the following features: proportionality, comprehensiveness, harm mitigation, exemptions for specific patient populations, visitation decisions made separately from a patient’s treating clinicians, transparency, and consistency in application” (p. 715). They also “argue that an ethical policy ought to include scope for case-by-case exemptions for individual patients” (p. 715). Notably, these same authors (32) created a flowchart to depict how choices, potentially by policymakers, should be made about exceptions to the “no visitors” policy, which may be applied during future crises. This flowchart included considering, first, the benefits and harms of allowing the exception (including the risk of spreading COVID-19, emotional impact, contribution to care, and cultural considerations). Next, the flowchart includes assessing alternative pathways to achieving the determined benefits and thwarting the risk of the spread of COVID-19. Finally, considerations are made for evaluating if the benefits outweigh the risks.

From the current study, it is recommended that evidence-based literature and stakeholders, including patients, caregivers, and organizations with expertise on caregiver inclusion in healthcare, should be consulted prior to implementing any measure or policy that may impede caregivers’ ability to engage in patient care and on healthcare teams. This includes building on approaches used in some health settings in Canada, including the Saskatchewan Health Authority and Kingston Health Sciences Centre in Ontario (64). It is also important to recognize that visitor restriction policy requires resources to implement, which would be another dimension to consider in the face of resource demands during a pandemic.

### Strengths and limitations of the study

This study provides a robust qualitative examination of the additional work required of HCPs to navigate and manage the “no visitors” policy throughout the pandemic. There are several strengths to acknowledge. Namely, the transferability of findings is supported because the authors were able to provide an analysis of similar findings across two independent studies of different factions of healthcare, concerning different provider, patient, and caregiver populations, across different hospital settings. In this regard, the authors have provided a diversity of perspectives on the many impacts of visitor restrictions from a sizable qualitative sample. Nevertheless, there are several limitations to acknowledge. While a diverse and sizable overall sample, most participants were female, and a relatively small proportion of interviews were conducted with caregivers themselves. Despite the value of caregiver perspectives and our efforts to recruit further, this paper may have benefited from the inclusion of more caregivers and patients, enabling us to further understand the consequences of top-down rapid policy generation that largely failed to include these perspectives. It may also be prudent for future work to develop explicit strategies to recruit a more sex- and gender-diverse population to better incorporate more diversity in social location. Interviews were also conducted primarily after the initial peaks in intensity of the pandemic but still occurred throughout the latter stages. This does not, however, diminish the value of these findings as the impacts of pandemic-based policy generation and implementation continue to shape healthcare system functioning in profound ways. Findings such as these may serve to better inform future decision-making processes around visitor restriction policies in healthcare and beyond. Future research to further support decision-making regarding visitor restrictions should draw attention to co-designed, sustainable, and equitable alternatives for connecting patients with loved ones (distinguished from caregivers, who have been demonstrated as essential to care) when infection control precautions are necessary for public health safety.

## Conclusion

This qualitative research study explored the “no visitors” policy implemented during the COVID-19 pandemic in Ontario, Canada, and in particular, the work to manage and work around these limitations across healthcare system silos, focusing on cancer care and ALC transitions in care. Overall, managing the “no visitors” policy and pushback against it generated a significant burden of work for patients, caregivers, healthcare providers, and healthcare decision-makers, and participants had to engage in burdensome work to manage the restrictions. Not allowing caregivers into hospitals during the COVID-19 pandemic signaled that their work was devalued and that they were not *really* viewed as imperative to patient care nor the health care team (27, 70).

Lessons learned from the implementation of the “no visitors” policy point to the importance of caregivers being treated as vital members of healthcare teams; a tenet supported by other work (20, 22, 71–73) given caregivers’ importance to patient safety, building trust, and to person- and family-centered care (22, 48, 49). The evidence for harms resulting from the implementation of strict “no visitors” restrictions is growing and suggests that closer attention and care to research detailing the unintended consequences of such public health policy is required.

## Data Availability

The datasets presented in this article are not readily available because of participants’ right to anonymity and confidentiality. The anonymized raw data contains details from participants’ experiences, which may allow for identification by familiar individuals. All data relevant to the thematic analysis is included in the findings section of the manuscript. Requests to access the datasets should be directed to danielle.jacobson@thp.ca.
